# Supplementary material for: Risk relationship between inflammatory bowel disease and urolithiasis: A two-sample Mendelian randomization study
Source: PLoS One. 2024 Apr 9;19(4):e0301545. doi: 10.1371/journal.pone.0301545 (PMC11003619; doi:10.1371/journal.pone.0301545)
Supplement: Attachment — Submitted filename: Response to Reviewers.docx [file pone.0301545.s001.docx]

Dear Editor,

We feel great thanks for your professional review work on our article. As you are concerned, there are several problems that need to be addressed. According to your suggestions, we have made extensive corrections to our previous draft, the detailed corrections are listed below:

1.We have tried our best to modify the format of the article in accordance with the style requirements of PLOS ONE's, which is mainly reflected in terms of titles at all levels, references and file naming requirements, etc.

2.Thank you for your reminding. We have made some improvements such as text weight reduction and sentence modification for repeated text. Specific changes in the “Revised Manuscript with Track Changes” are highlighted in yellow.

3.Thanks for your reminding, we have reviewed and modified the quoted source once again. At the same time, we have also rephrase duplicated text outside the methods.

4. We have corrected as much as possible the language use, spelling and grammar problems that may have appeared in the previous manuscript.
5. Thank you for your suggestions, and changes specific to the funding statement will be attached to the resubmitted cover letter.
6. We have carefully reviewed and revised the references in the article, and the specific modifications are as follows:
1) The number of authors shown in references was revised according to the style requirements of PLOS ONE. (references 5-6, 8-9,11,13,16,18,19,22,28,39,40)
2) For references 12 and 36, considering the published year too long possible adaptation degree is not high, we replaced it.
3) Controversial or inappropriate references such as 24,26,31 have been replaced to better fit the quotation.

The above modifications are reflected in the file "Revised Manuscript with Track Changes".

Dear reviewer,

We would also like to thank all reviewers for their valuable feedback that we have used to improve the quality of our manuscript. The reviewer comments are laid out below in italicized font and specific concerns have been numbered. Our response is given in normal font.

***Reviewer #1:***

*I have limited knowledge of the statistical methods used. With this limitation, my comments are:*

1) *I do not understand what the three study groups (with their individual controls) are: apart from Crohn's disease and ulcerative colitis, there is a third group of "IBD". What is this third group?*

**The author’s answer:** Thank you for raising this question. We found some errors in the description of this question in the manuscript. The current study used a two-sample, multivariate Mendelian randomization analysis. Crohn's disease(CD) and ulcerative colitis(UC) are subtypes of inflammatory bowel diseases(IBD). In our study, two subgroups were analyzed, and the results were detailed in Result 3.1. Accordingly, we modified the ambiguous description, as shown in lines 194-201 of the manuscript.

2) *The OR is just above 1.00 in these cases. If I understand right, this means the risk of coexistence is only slightly above expected what is expected from the general population.*

**The author’s answer:** Thank you for your comments. As you said, the OR results in this study only indicate a low risk correlation, but the phenomenon that IBD is less clear about the specific risk factors of urolithiasis, we think the analysis results are still of certain significance.

3) *The Introduction is too lengthy and simplistic. This can be made more precise and focused.*

**The author’s answer:** Thank you for your suggestions. We have cut and modified the long or repetitive narrative statements in a targeted manner.

4) *The authors use a lot of introductory phrases and adjectives, which can be deleted during editing.*

**The author’s answer:** Thank you for your comments, and we have also made some modifications in the resubmitted manuscript.

***Reviewer #2:***

1) *The association of inflammatory Intestinal Bowel disease and renal involvement is well known – the most frequent renal diseases are: nephrolithiasis, tubulointerstial nephritis, glomerulonephritis and amyloidosis. As an example, PubMED for “renal lithiasis and inflammatory bowel disease” retrieved 147 results.*

**The author’s answer:** Thank you for providing the conclusion of the correlation search on the relationship between inflammatory bowel disease and renal involvement. We included the information you provided in the introduction section of the manuscript. Lines 86 to 89 are shown in the manuscript.

2) *The authors used the Mendelian Randomization Analysis. This method has been criticized as follows:
   “In recent years, epidemiologists have increasingly sought to employ genetic data to identify ‘causal’ relationships between exposures of interest and various endpoints – an instrumental variable approach sometimes termed Mendelian randomization.*
   *However,* *this approach is subject to all of the limitations of instrumental variable analysis and to several limitations specific to its genetic underpinnings, including confounding, weak instrument bias, pleiotropy, adaptation, and failure of replication.*
   *Although the approach enjoys some utility in testing the etiological role of discrete biochemical pathways, like folate metabolism, this method must be treated with all the circumspection that should accompany all forms of observational epidemiology.
   Going forward, we urge the elimination of* *randomization or causality in reports of its use and suggest that Mendelian randomization instead be termed exactly what it is – genetic instrumental variable analysis”.
   (Kenneth J Mukamai, Meir J.Stampfer and Eric B.Rimm; European Journal of Epidemiology, volume 35, pages 93-97 (2020).
   I suggest that this article should be evaluated also by a Statistics Expert.*

**The author’s answer:** Thank you for supplementing the literature on the methodological limitations of Mendelian randomization studies. We thought this would provide greater credibility to our manuscript, and therefore, we cited this literature to lines 341-346 in the strengths and limitations section of the manuscript.

Thank you very much for your attention and time. Look forward to hearing from you.

Yours sincerely,

Xue-lin Jin

22-Dec., 2023

Panzhihua University, Affiliated Hospital, Anorectal
