# Supplementary material for: Risk relationship between inflammatory bowel disease and urolithiasis: A two-sample Mendelian randomization study
Source: PLoS One. 2024 Apr 9;19(4):e0301545. doi: 10.1371/journal.pone.0301545 (PMC11003619; doi:10.1371/journal.pone.0301545)
Supplement: Attachment — Submitted filename: Response to Reviewers.docx [file pone.0301545.s002.docx]

Dear Editor,

We feel great thanks for your professional review work on our article again. As you are concerned, there are several problems that need to be addressed. Questions are laid out below in italicized font and specific concerns have been numbered. Our response is given in normal font.

1. *Please review your reference list to ensure that it is complete and correct. If you have cited papers that have been retracted, please include the rationale for doing so in the manuscript text, or remove these references and replace them with relevant current references. Any changes to the reference list should be mentioned in the rebuttal letter that accompanies your revised manuscript. If you need to cite a retracted article, indicate the article’s retracted status in the References list and also include a citation and full reference for the retraction notice.*
   **The author’s answer:** We did our best to search the original text of the referenced articles, but we did not get accurate retraction information for the cited articles, such as being marked as "retracted". Therefore, we were only able to make changes to references that were subjectively controversial in our judgment. Thank you for your comments. If possible, we hope you can provide us with more precise suggestions on this issue. The specific modifications are as follows:
1) For references 4 (Romero V, Akpinar H, Assimos DG. Kidney stones: a global picture of prevalence, incidence, and associated risk factors. Rev Urol. 2010;12(2– 3):e86–e96.), there may have been insufficient matching of citation relevance, which has now been replaced with more convincing literature citations.
2) For references 15 (Katan MB. Apolipoprotein E isoforms, serum cholesterol, and cancer. Lancet. 1986 ;1(8479):507–508), it was replaced with “Davey Smith G, Hemani G. Mendelian randomization: genetic anchors for causal inference in epidemiological studies. Hum Mol Genet. 2014 Sep 15;23(R1):R89-98.”.
3) For references 33 (Dröge W. Free radicals in the physiological control of cell function. Physiol Rev. 2002;82(1):47–95.), we replaced it with more convincing reference “Liu Y, Sun Y, Kang J, He Z, Liu Q, Wu J, et al. Role of ROS-Induced NLRP3 Inflammasome Activation in the Formation of Calcium Oxalate Nephrolithiasis. Front Immunol. 2022 Jan 27;13:818625.”.
4) Reference 5, as an article published in 1986, may have retrieval problems because of its remote age, but this article is one of the earliest studies that can be traced back to the association between inflammatory bowel disease and kidney stones. If this reference does not meet the requirements, we may replace it or remove it.
2. *Abstract: how many* *patient samples and how many SNPs were examined? It is unclear from this text, and seems perhaps contradictor to later in the paper where the 3 sets of controls are mentioned.*

**The author’s answer:**

About the patient samples: The dataset used in this study included a total of four groups of relevant samples, specific single nucleotide polymorphism data were obtained from GWASs, including IBD (n=59957, 25,042 IBD cases and 34,915 controls) and its main subtypes, Crohn's disease (CD) (n=40266, 12,194 CD cases and 28,072 controls) and ulcerative colitis (UC) (n=45975, 12,366 UC cases and 33,609 controls). Summarized data on urolithiasis (n=218792, 5,347 urolithiasis cases and 213445 controls) were obtained from different GWAS studies from FINNGEN database. The specific changes are in lines 27 and 121 of the manuscript.

About SNPs: In this study, we aimed to investigate the causal relationship between inflammatory bowel disease (and its two subtypes, Crohn's disease and ulcerative colitis) and urolithiasis using a two-sample MR approach. Three sets of genetic instruments were utilized in the analysis, all drawn from publicly available genetic summary data. The first set consisted of 259 single-nucleotide polymorphisms (SNPs) robustly linked to inflammatory bowel disease (IBD), the second set was comprised of 81 SNPs associated with inflammatory bowel disease (CD) and the third set consisted of 175 SNPs associated with ulcerative colitis (UC). If necessary, we will be able to provide the corresponding raw data sets.

3. *Is the manuscript presented in an intelligible fashion and written in standard English?*

**The author’s answer:** We suggest you thoroughly copyedit your manuscript for language usage, spelling, and grammar. If you do not know anyone who can help you do this, you may wish to consider employing a professional scientific editing service.

We revised the language use, spelling, and grammar in the manuscript again as suggested by you. We also employed a professional scientific editor from Editage to assist us in revising the manuscript for publication in Plos one.

Thank you very much for your attention and time. Look forward to hearing from you.

Yours sincerely,

Xue-lin Jin

5-Mar., 2024

Panzhihua University, Affiliated Hospital, Anorectal
